# A Case Study of Discordant Overlapping Meta-Analyses: Vitamin D Supplements and Fracture

**DOI:** 10.1371/journal.pone.0115934

**Published:** 2014-12-31

**Authors:** Mark J. Bolland, Andrew Grey

**Affiliations:** Department of Medicine, University of Auckland, Private Bag 92 019, Auckland 1142, New Zealand; Children's National Medical Center, Washington, United States of America

## Abstract

**Background:**

Overlapping meta-analyses on the same topic are now very common, and discordant results often occur. To explore why discordant results arise, we examined a common topic for overlapping meta-analyses- vitamin D supplements and fracture.

**Methods and Findings:**

We identified 24 meta-analyses of vitamin D (with or without calcium) and fracture in a PubMed search in October 2013, and analysed a sample of 7 meta-analyses in the highest ranking general medicine journals. We used the AMSTAR tool to assess the quality of the meta-analyses, and compared their methodologies, analytic techniques and results. Applying the AMSTAR tool suggested the meta-analyses were generally of high quality. Despite this, there were important differences in trial selection, data extraction, and analytical methods that were only apparent after detailed assessment. 25 trials were included in at least one meta-analysis. Four meta-analyses included all eligible trials according to the stated inclusion and exclusion criteria, but the other 3 meta-analyses “missed” between 3 and 8 trials, and 2 meta-analyses included apparently ineligible trials. The relative risks used for individual trials differed between meta-analyses for total fracture in 10 of 15 trials, and for hip fracture in 6 of 12 trials, because of different outcome definitions and analytic approaches. The majority of differences (11/16) led to more favourable estimates of vitamin D efficacy compared to estimates derived from unadjusted intention-to-treat analyses using all randomised participants. The conclusions of the meta-analyses were discordant, ranging from strong statements that vitamin D prevents fractures to equally strong statements that vitamin D without calcium does not prevent fractures.

**Conclusions:**

Substantial differences in trial selection, outcome definition and analytic methods between overlapping meta-analyses led to discordant estimates of the efficacy of vitamin D for fracture prevention. Strategies for conducting and reporting overlapping meta-analyses are required, to improve their accuracy and transparency.

## Introduction

The number of meta-analyses published in recent years has dramatically increased [1], [2]. Partly, this is because systematic reviews and meta-analyses are considered the highest level of evidence, but it is also relatively easy to undertake and publish a meta-analysis [3]. However, many meta-analyses are not novel, and either reproduce or extend earlier analyses on the same topic- i.e. are overlapping. In a random sample of meta-analyses that were published in 2010 and included randomised trials, 67% had at least one other overlapping meta-analysis [3]. Overlapping meta-analyses may report discordant results and conclusions, particularly as the number of such analyses increases. The consequences of this include contradictory recommendations for clinical practice, confusion amongst clinicians and their patients, and public disenchantment with clinical science.

Discordant meta-analyses have been reported previously for a variety of interventions [4]–[8], and recommendations for assessing such meta-analyses are available [9]. These recommendations focus on the methods and quality of the review, both of which have become much more standardised since the recommendations were proposed. The effect of vitamin D supplements on fracture is the subject of a large number of meta-analyses [10]. In 2012, an individual patient data meta-analysis was the 21^st^ meta-analysis published on this topic, but identified only 14 relevant randomised controlled trials [11]. Two recent clinical guidelines on vitamin D [12], [13], based on meta-analyses of the same clinical trials by independent groups, reached very different conclusions [14], [15]. As a case study of overlapping meta-analyses, we conducted a detailed review of meta-analyses of vitamin D and fracture. We investigated differences between the meta-analyses by applying recommendations for comparing discordant overlapping meta-analyses. We focused on the quality and methodology of the meta-analyses with regard to trials included, trial data utilised, analytic approaches, and conclusions, and considered the implications these differences have for clinical practice, interpretation of existing meta-analyses and performance of future analyses.

## Methods

### Ethics statement

Ethical approval was not required for this work.

We searched PubMed in October 2013 for systematic reviews and meta-analyses of vitamin D with fracture as an outcome (S1 Appendix). We identified 24 meta-analyses, and analysed the most recent meta-analysis in each of the highest ranking general medical journals (Ann Intern Med, BMJ, Cochrane Database Syst Rev, JAMA, JAMA Intern Med, Lancet, NEJM). We chose this sample of meta-analyses because they are likely to have been conducted to the highest standard, as well as being the most closely scrutinised during peer review and post-publication. Thus, we analysed 5 trial-level and 2 patient-level meta-analyses on the effect of vitamin D with or without calcium on fracture [16]–[22].

Jadad and colleagues recommended assessing discordant systematic reviews in 6 domains- the clinical question asked, study selection and inclusion, data extraction, study quality, ability to combine studies, and statistical methods [9]. Following this approach, the quality of each meta-analysis was assessed using the AMSTAR tool [23], and the inclusion and exclusion criteria, endpoints, trials included, and data on hip and total fracture outcomes for each contributing study were extracted by one author (MB) and checked by a second (AG). Differences were resolved by consensus. Some trials reported data for non-vertebral fracture rather than total fracture. In this situation, we used data for non-vertebral fracture when total fracture data were not available. Only one meta-analysis described the reasons for exclusion of individual trials in detail [18]. For each of the other meta-analyses, we assessed whether trials that were not included in the meta-analysis were eligible for inclusion according to the published inclusion and exclusion criteria, and tried to determine why the trial was not included. Where data for the efficacy of vitamin D on fracture outcomes for a trial differed between meta-analyses, we tried to determine the reason. The recommended approach to analysis of a randomised controlled trial is an unadjusted intention-to-treat analysis using all randomised participants with data from the final study timepoint [24]. We considered the result from this approach to be the best estimate of treatment efficacy. An analysis restricted to those participants who completed the study was termed a “per-protocol analysis.” Finally, we compared the conclusions of the meta-analyses, with each author independently rating the strength of the conclusions toward the use of vitamin D supplementation to prevent fracture on a three point scale (positive/mixed/negative toward vitamin D supplementation) and on a scale from 1 to 5 (1 most negative, 5 most positive toward vitamin D supplementation). These ratings were based solely on the conclusions of the meta-analysis, and did not consider the data or analyses used in the meta-analyses.

## Results

Table 1 shows the characteristics of the 7 meta-analyses and the trials included in each meta-analysis. The number of included trials in each meta-analysis ranged from 7 to 20. Of the 25 trials included in any of the meta-analyses [25]–[50], 6 were included in only 1 meta-analysis, 3 in 2 meta-analyses, 2 in 3 meta-analyses, 3 in 4 meta-analyses, 7 in 5 meta-analyses, and 4 in 6 meta-analyses. No trial was included in all meta-analyses. The number of trials that met criteria for inclusion but were not included in each meta-analysis ranged from 0 to 8 trials: 4 meta-analyses included all trials that met the eligibility criteria [16]–[18], [20], with 2 meta-analyses “missing” 3 trials [19], [22], and 1 missing 8 trials [21]. Of the 8 trials that were missing from at least 1 meta-analysis, 4 were missed in 1 meta-analysis, 2 in 2 meta-analyses, and 2 in 3 meta-analyses. Two meta-analyses [16], [17] included 1 and 3 trials, respectively, that did not appear to meet the stated eligibility criteria (Table 1). In both cases, other trials were not included in the meta-analyses despite having similar design to the included trials that appeared ineligible.

**Table 1:** Characteristics of 7 included meta-analyses and trials included or excluded in each meta-analysis.

Author	Bischoff-Ferrari, 2005 [16]	Tang, 2007 [17]	Bischoff-Ferrari, 2009 [19]	Avenell, 2009 [18]	DIPART, 2010 [20]	Chung, 2011 [21]	Bischoff-Ferrari, 2012 [22]
**Level of data**	Trial	Trial	Trial	Trial	Patient	Trial	Patient
**Search end date**	Jan 2005	Jan 2007	Aug 2008	Sept 2007	July 2008	July 2011	Aug 2011
**Inclusion criteria**	Vit D+/− calcium	Vit D+ calcium	Vit D+/− calcium	Vit D+/− calcium	Vit D+/− calcium	Vit D+/− calcium	Vit D+/− calcium
	Double-blind trials	Placebo-controlled trials	Oral supplements	Men>65	N≥1000	>1 month	Oral supplements
	Oral supplements	Age ≥50 y	≥1 year	Postmenopausal women			Age ≥65 y
	≥1 year		≥1 fracture				
	≥1 fracture		Mean age ≥65 y				
	Mean age>60 y						
**Exclusion criteria**	Major morbidity	Secondary osteoporosis	Major morbidity	Glucocorticoids	Data censored	Pregnancy	Untreated controls
				Factorial studies	at 36 m	Major morbidity	
				(except calcium)			
**Endpoints (fracture)**	Hip	Total	Non-vertebral	Hip	Total	Total	Hip
	Non-vertebral		Hip	Non-vertebral	Hip		Non-vertebral
				Vertebral			
				Total			
**Included Trials**							
Chapuy 1992/1994 [25], [26]	Yes	Yes	Yes	Yes	X- NDA	Yes	Yes
Lips 1996 [27]	Yes	X-agent	Yes	Yes	X- NDA	Yes	Yes
Dawson-Hughes 1997 [28]	Yes	Yes	Yes	Yes	X-size	Yes	Yes
Komulainen 1998 [29]	X-age	X-agent	X-age	Yes	X-size	Yes	X-age
Pfeifer 2000 [30]	Yes	X-agent	Yes	Yes	X-size	Yes	Yes
Chapuy 2002 [31]	Yes	Yes	Yes	Yes	X-size	Yes	X-NDA
Meyer 2002 [32]	Yes	X-agent	Yes	Yes	Yes	X-uncertain	Yes
Bischoff 2003 [33]	X-duration	X-agent	X-duration	Yes	X-size	X-uncertain	X-uncertain
Trivedi 2003 [34]	Yes	X-agent	Yes	Yes	X- NDA	Yes	X-NDA
Avenell 2004 [35]	X-design	X-design	X-uncertain^a^	Yes	X-size	X-uncertain^a^	X-controls
Harwood 2004 [36]	X-design	Yes^b^	Secondary	Yes	X-size	Yes	X-controls
Larsen 2004 [37]	Secondary^b^	Yes^b^	Secondary	X-design	Yes	X-uncertain	X-controls
Flicker 2005 [38]	Secondary	X-agent	Yes	Yes	X-size	Yes	Yes
Grant 2005 [39]	X-date	Yes	Yes	Yes	Yes	Yes	Yes
Law 2006 [40]	X-date	X-agent	Secondary	Yes	X- NDA	Yes	X-controls
Jackson 2006 [41]	X-date	Yes	Yes	Yes	Yes	Yes	Yes
Porthouse 2006 [42]	X-date	Yes^b^	Secondary	Yes	Yes	Yes	X-controls
Bolton-Smith 2007 [43]	X-date	X-date	X-uncertain^a^	Yes	X-size	X-uncertain^a^	X-uncertain^a^
Lyons 2007 [44]	X-date	X-date	Yes	Yes	Yes	Yes	Yes
Smith 2007 [45]	X-date	X-date	X-IM	Yes	Yes	X-uncertain	X-IM
Prince 2008 [46]	X-date	X-date	X-uncertain	Yes	X-size	X-uncertain	X-uncertain
Pfeifer 2009 [47]	Secondary	X-date	Yes	X-date	X-date	X-uncertain	Yes
Bischoff-Ferrari 2010 [48]	X-date	X-date	X-date	X-date	X-date	X-design	Yes
Salovaara 2010 [49]	X-date	X-date	X-date	X-date	X-date	Yes	X-controls
Sanders 2010 [50]	X-date	X-date	X-date	X-date	X-date	Yes	Secondary

X =  study not included in meta-analysis. Reasons for non-inclusion: NDA- eligible for inclusion but no patient-level data available; agent- did not compare vitamin D plus calcium with placebo; size- study smaller than inclusion criteria allowed; age- age outside inclusion criteria; uncertain- unknown reason for exclusion; duration- duration of study less than inclusion criteria allowed; design- design did not meet inclusion criteria; controls- untreated control group; date- after search date; IM- intramuscular administration.

a limited or no fracture data in primary publication, but data obtained from lead author and published in at least 1 meta-analysis. The Bolton-Smith fracture trial data were not published in Avenell 2009 until after publication of the Bischoff-Ferrari 2009 meta-analysis.

b trial does not appear to meet eligibility criteria for meta-analysis.

Abbreviations: Secondary- included in secondary analyses only. Vit D: vitamin D.

Table 2 shows the quality assessment of each meta-analysis. Generally, the meta-analyses were of high quality, although all meta-analyses did not report some of the AMSTAR items, and some of the methods used in 3 meta-analyses [19], [21], [22] were of uncertain validity. Reporting of AMSTAR items was less common in the 2 patient-level meta-analyses [20], [22].

**Table 2:** Quality assessment of meta-analyses.

AMSTAR item [23]	Bischoff-Ferrari, 2005 [16]	Tang, 2007 [17]	Bischoff-Ferrari, 2009 [19]	Avenell, 2009 [18]	DIPART, 2010 [20]	Chung, 2011 [21]	Bischoff-Ferrari, 2012 [22]
1. A priori design	Yes	Yes	Yes	Yes	Yes	Yes	Yes
2. Duplicate study selection	Not stated	Not stated	Not stated	Yes	Not stated	Not stated	Not stated
2. Duplicate data extraction	Yes	Yes	Yes	Yes	Not applicable	Yes	Not applicable
3. Comprehensive literature search	Yes	Yes	Yes	Yes	No^a^	No^a^	No^a^
4. Status of publication used as an inclusion criterion	No	No	No	No	No	Yes	No
5. List of included studies	Yes	Yes	Yes	Yes	Yes	Yes	Yes
5. List of excluded studies	No	No	No	Yes	No	No	No
6.Characteristics of included studies reported	Yes	Yes	Yes	Yes	Yes	Yes	No
7. Quality of studies assessed	Yes	Yes	Yes	Yes	No	Yes	No
8. Quality of studies used appropriately in formulating conclusions	Yes	Yes	Yes	Yes	No	Yes	No
9. Appropriate methods used to combine results	Yes	Yes	No^b^	Yes	Yes	No^c^	No^d^
10. Publication bias assessed	Yes	Yes	Yes	Yes	No	No	No
11. Conflict of interests noted for review	Yes	Yes	Yes	Yes	Yes	Yes	Yes^e^
11. Conflict of interests noted for included studies	No	No	No	No	No	No	No

a No search for grey literature described.

b Data combined appropriately using random-effects models, but studies grouped according to received dose (treatment dose * adherence). The advisability and validity of this approach is uncertain.

c Data combined appropriately using random-effects models, but data for hip fracture was used for 4/16 trials when the primary endpoint assessed was total fracture.

d Data combined appropriately using Cox proportional-hazards models, but method of assessment of vitamin D intake differed between treatment and control groups.

e Partly funded by a manufacturer of vitamin D supplements.

Tables 3 and 4 show data from each trial used in each meta-analysis for hip fracture and total fracture, respectively. For hip fracture, the relative risk differed between meta-analyses for 6 of 12 trials for which data were reported in two or more meta-analyses, and for total fracture, the relative risk differed between meta-analyses in 10 of 15 trials. Tables 3 and 4 show the reasons for the differences in relative risks between the meta-analyses, which are summarised in Table 5. Many of the differences arose when results obtained using analyses other than the recommend approach (unadjusted intention-to-treat analysis of all participants with data from the final study timepoint) were used in a meta-analysis, with the most common example being the use of a per-protocol analysis. The majority of the differences (4 of 6 for hip fracture, 7 of 10 for total fracture) led to more favourable estimates of the efficacy of vitamin D on hip or total fracture being used for individual trials than if the recommended approach was applied. In general, the Cochrane review [18] was most likely to use the recommended approach, and the relative risks used for each study in that review are the most conservative.

**Table 3:** Data for hip fracture in each meta-analysis.

	Bischoff-Ferrari 2005 [16]		Bischoff-Ferrari 2009 [19]		Avenell 2009 [18]		Chung 2011 [21]	
	Vit D	Controls	Vit D	Controls	Vit D	Controls	Vit D	Controls
	(n/N)	(n/N)	(n/N)	(n/N)	(n/N)	(n/N)	(n/N)	(n/N)
**Trial**	RR (95% CI)		RR (95% CI)		RR (95% CI)		RR (95% CI)	
**Chapuy, 1992/1994 ** [25], [26]	137/1176	178/1127	137/1176	178/1127	137/1634	178/1636	80/1387	110/1403
	**0.74 (0.60–0.91)^b,^** c		**0.738 (0.6–0.907)^b,^** c		**0.77 (0.62–0.95)^b^**		**0.74 (0.56–0.97)** c **^,^** d	
**Lips, 1996 ** [27]	58/1291	48/1287	58/1291	48/1287	58/1291	48/1287	58/1291	48/1287
	1.21 (0.83–1.75)		1.205 (0.829–1.751)		1.20 (0.83–1.75)		1.20 (0.83–1.75)	
**Dawson-Hughes, 1997 ** [28]	0/187	1/202	Data not included		0/187	1/202	Data not included	
	NS	NS						
**Pfeifer, 2000 ** [30]	0/70	1/67	Data not included		Data not included		Data not included	
	NS	NS						
**Chapuy, 2002 ** [31]	27/393	21/190	27/393	21/190	27/389	21/194	27/393	21/190
	**0.62 (0.36–1.07)** e		**0.622 (0.362–1.068)** e		**0.64 (0.37–1.10)**		**0.62 (0.36–1.07)** e	
**Meyer, 2002 ** [32]	50/569	47/575	50/569	47/575	50/569	47/575	Study not included	
	1.08 (0.73–1.57)		1.075 (0.734–1.574)		1.08 (0.73–1.57)			
**Bischoff, 2003 ** [33]	Study excluded		Study excluded		2/62	1/60	Study not included	
					1.94 (0.18–20.79)			
**Trivedi, 2003 ** [34]	21/1345	24/1341	21/1345	24/1341	21/1345	24/1341	Data not included	
	**0.85 (0.47–1.53)** f		**0.85 (0.47–1.53)** f		**0.87 (0.49–1.56)**			
**Avenell, 2004 ** [35] a	Study excluded		Study not included				Study not included	
Vit D vs placebo					0/35	1/35		
					0.33 (0.01–7.91			
CaD vs placebo					1/35	1/35		
					1.00 (0.07–15.36)			
**Harwood, 2004 ** [36] a	Study excluded		Study not included					
Vit D vs placebo					0/38	1/37	3/39	5/37
					**0.32 (0.01–7.73)**		**0.57 (0.15–2.22)** g	
CaD vs placebo					1/75	1/37		
					0.49 (0.03–7.67)			
**Grant, 2005 ** [39] a	Study excluded						Data not included	
Vit D vs placebo					47/1343	41/1332		
					1.14 (0.75–1.72)			
CaD vs placebo					46/1306	41/1332		
					1.14 (0.76–1.73)			
Vit D vs no Vit D			93/2649	90/2643				
			1.031 (0.776–1.371)					
**Law, 2006 ** [40]	Study excluded		24/1762	20/1955	17/1252	14/1389	24/1762	20/1955
			**1.331 (0.740–2.397)**		**1.35 (0.67–2.72)** h		**1.33 (0.74–2.40)**	
**Jackson, 2006 ** [41]	Study excluded		146/11448	186/11412	175/18176	199/18106	Data not included	
			**0.782 (0.631–0.970)** i		**0.88 (0.72–1.07)**			
**Porthouse, 2006 ** [42]	Study excluded		8/1321	17/1993	8/1321	17/1993	Data not included	
			0.710 (0.309–1.633)		0.71 (0.31–1.64)			
**Lyons, 2007 ** [44]	Study excluded		112/1725	104/1715	112/1725	104/1715	Data not included	
			1.071 (0.827–1.386)		1.07 (0.83–1.39)			
**Smith, 2007 ** [45]	Study excluded		Study excluded		66/4727	44/4713	Study not included	
					1.50 (1.02–2.19)			

Data for hip fracture were not analysed in Tang 2007 [17], and individual trial data for hip fracture were not reported in DIPART 2010 [20], or Bischoff-Ferrari 2012 [22]. No data on hip fractures were reported in any meta-analysis for Komulainen 1998 [29], Larsen 2004 [37], Flicker 2005 [38], Bolton-Smith 2007 [43], Prince 2008 [46], Pfeifer 2009 [47], Bischoff-Ferrari 2010 [48], Salovaara 2010 [49], and Sanders 2010 [50].

Study excluded- study met meta-analysis exclusion criteria. Study not included- study did not meet exclusion criteria but not included in meta-analysis. Data not included- data not included in meta-analysis but available from other meta-analyses or primary publication.

Abbreviations: Vit D- vitamin D, RR- relative risk, CI confidence interval. NS- not stated. Vit D- vitamin D. CaD- co-administered calcium and vitamin D.

Bold text- indicates differences in relative risks for individual studies between meta-analyses.

a factorial/multi-arm studies permitting multiple comparisons between randomised groups.

Reasons for differences in reported data between meta-analyses: ^b^data after 36 month from Chapuy 1994;

c per-protocol analysis (all randomised participants not included);

d data after 18 month from Chapuy 1992;

e original paper reports different numbers for placebo group, correct numbers confirmed by original authors in Avenell 2009;

f age-adjusted relative risk;

g data for total fracture not hip fracture;

h adjustments to the number of participants with outcomes and denominators were made using an intraclass correlation coefficient of 0.026 to account for cluster randomisation;

i subgroup of participants only.

**Table 4:** Data for total or non-vertebral fracture in each meta-analysis.

	Bischoff-Ferrari 2005 [16]		Tang 2007 [17]		Bischoff-Ferrari 2009 [19]		Avenell 2009 [18]		Chung 2011 [21]	
	Vit D	Controls	Vit D	Controls	Vit D	Controls	Vit D	Controls	Vit D	Controls
	(n/N)	(n/N)	(n/N)	(n/N)	(n/N)	(n/N)	(n/N)	(n/N)	(n/N)	(n/N)
**Trial**	RR (95% CI)		RR (95% CI)		RR (95% CI)		RR (95% CI)		RR (95% CI)	
**Chapuy, 1992/1994 ** [25], [26]	255/1176	308/1127	NS/NS	NS/NS	255/1176	308/1127	255/1634	308/1636	Data not included	
	**0.79 (0.69–0.92)^b,^** c **^,^** d		**0.75 (0.64–0.87)^b,^** d **^,^** e **^,^** f		**0.793 (0.687–0.916)^b,^** c **^,^** d		**0.83 (0.71–0.96)^b,^** c			
**Lips, 1996 ** [27]	135/1291	122/1287	Study excluded		135/1291	122/1287	135/1291	122/1287	Data not included	
	1.10 (0.87–1.39)^b^									
**Dawson-Hughes, 1997 ** [28]	11/202	26/187	NS/NS	NS/NS	11/187	26/202	11/187	26/202	11/187	26/202
	0.46 (0.24–0.88)^b^		0.46 (0.23–0.90)^b^		0.457 (0.237–0.879)^b^		0.46 (0.23–0.90)^b^		0.46 (0.23–0.90)^b^	
**Komulainen, 1998 ** [29]	Study excluded		Study excluded		Study excluded		11/116	15/116	8/116	14/116
							**0.73 (0.35–1.53)^b^**		**0.57 (0.25–1.31)^b,^** d	
**Pfeifer, 2000 ** [30]	3/70	6/67	Study excluded		3/70	6/67	3/74	6/74	3/70	6/67
	**0.48 (0.13–1.78)^b,^** d				**0.479 (0.129–1.782)^b,^** d		**0.50 (0.13–1.92)^b^**		**0.48 (0.12–1.84)^b,^** d	
**Chapuy, 2002 ** [31]	97/393	55/190	NS/NS	NS/NS	97/393	55/190	69/389	34/194	Data not included	
	**0.85 (0.64–1.13)^b,^** f **^,^** g		**0.85 (0.64–1.13)^b,^** f **^,^** g		**0.853 (0.642–1.133)^b,^** f **^,^** g		**1.01 (0.70–1.47)^b^**			
**Meyer, 2002 ** [32]	69/569	76/575	Study excluded		69/569	76/575	69/569	76/575	Study not included	
	0.92 (0.68–1.24)^b^				0.917 (0.677–1.244)^b^		0.92 (0.68–1.24)^b^			
**Trivedi, 2003 ** [34]	43/1345	62/1341	Study excluded		43/1345	62/1341	119/1345	149/1341	119/1345	149/1341
	**0.67 (0.46–0.99)** h				**0.67 (0.46–0.99)** h		**0.80 (0.63–1.00)**		**0.80 (0.63–1.00)**	
**Avenell, 2004 ** [35] a	Study excluded		Study excluded		Study not included				Study not included	
Vit D vs placebo							3/35	5/35		
							0.60 (0.16–2.32)^b^			
CaD vs placebo							3/35	5/35		
							0.60 (0.16–2.32)^b^			
**Harwood, 2004 ** [36] a	Study excluded								Data not included	
Vit D vs placebo							0/38	5/37		
							0.09 (0.01–1.55)^b^			
CaD vs placebo			NS/NS	NS/NS	3/39	5/37	6/75	5/37		
			**0.49 (0.03–7.67)** i		**0.569 (0.148–2.185)^b,^** j		**0.59 (0.19–1.81)^b,^** k			
**Larsen, 2004 ** [37]	NS/NS	NS/NS	NS/NS	NS/NS	318/4957	167/2116	Study excluded		Study not included	
	NS		0.84 (0.72–0.98)^l^		NS^l^^,^ m					
**Flicker, 2005 ** [38]	NS/NS	NS/NS	Study excluded		25/313	35/312	25/313	35/312	25/313	35/312
	NS				0.712 (0.438–1.158)		0.71 (0.44–1.16)		0.71 (0.44–1.16)	
**Grant, 2005 ** [39] a	Study excluded									
Vit D vs placebo							188/1343	179/1332		
							1.04 (0.86–1.26)^n^			
CaD vs placebo			NS/NS	NS/NS			165/1306	178/1332		
			**0.94 (0.77–1.15)** n				**0.95 (0.78–1.15)** o			
Vit D vs no Vit D					349/2649	341/2643			387/2649	377/2643
					**1.021 (0.888–1.174)** p				**1.02 (0.90–1.17)**	
**Law, 2006 ** [40]	Study excluded		Study excluded		64/1762	51/1955	45/1252	36/1389	Data not included	
					**1.392 (0.971–1.997)^b^**		**1.39 (0.90–2.14)^b,^** q			
**Jackson, 2006 ** [41]	Study excluded		NS/NS	NS/NS	146/11448	186/11412	1921/18176	1961/18106	2102/18176	2158/18106
			**0.97 (0.92–1.03)**		**0.782 (0.631–0.970)** r **^,^** s		**0.98 (0.92–1.04)** t		**0.97 (0.92–1.03)**	
**Porthouse, 2006 ** [42]	Study excluded		NS/NS	NS/NS	58/1321	91/1993	58/1321	91/1993	58/1321	91/1993
			0.96 (0.70–1.33)		0.962 (0.697–1.327)		0.96 (0.70–1.33)		NS^u^	
**Bolton-Smith, 2007 ** [43]	Study excluded		Study excluded		Study not included		2/62	2/61	Study not included	
							0.98 (0.14–6.76)^b^			
**Lyons, 2007 ** [44]	Study excluded		Study excluded		202/1725	209/1715	205/1725	218/1715	243/1670	268/1673
					**0.961 (0.801–1.152)** t		**0.93 (0.78–1.12)**		**0.91 (0.77–1.07)** v	
**Smith, 2007 ** [45]	Study excluded		Study excluded		Study excluded		306/4727	279/4713	Study not included	
							1.09 (0.93–1.28)^b^			
**Prince, 2008 ** [46]	Study excluded		Study excluded		Study not included		4/151	3/151	Study not included	
							1.33 (0.30–5.86)			
**Pfeifer, 2009 ** [47]	NS/NS	NS/NS	Study excluded		9/121	16/121	Study excluded		Study not included	
	NS^b^				0.563 (0.262–1.208)^b,^ v					
**Salovaara, 2010 ** [49]	Study excluded		Study excluded		Study excluded		Study excluded		78/1586	94/1609
									0.84 (0.63–1.13)	
**Sanders, 2010 ** [50]	Study excluded		Study excluded		Study excluded		Study excluded		171/1131	135/1128
									1.26 (1.02–1.56)^v^	

Data are for total fracture unless otherwise indicated. Individual trial data for fracture were not reported in DIPART 2010 [20], or Bischoff-Ferrari 2012 [22]. No data on total or non-vertebral fracture were reported in any meta-analysis for Bischoff 2003 [33], and Bischoff-Ferrari 2010 [48].

Study excluded- study met meta-analysis exclusion criteria. Study not included- study did not meet exclusion criteria but not included in meta-analysis. Data not included- data not included in meta-analysis but available from other meta-analyses or primary publication.

Abbreviations: Vit D- vitamin D, RR- relative risk, CI confidence interval. NS- not stated. Vit D- vitamin D. CaD- co-administered calcium and vitamin D.

Bold text- indicates differences in relative risks for individual studies between meta-analyses.

a factorial/multi-arm studies permitting multiple comparisons between randomised groups.

Reasons for differences in reported data between meta-analyses: ^b^non-vertebral fracture,

c data after 36 month from Chapuy 1994;

d per-protocol analysis (all randomised participants not included);

e data after 18 month from Chapuy 1992;

f hip fractures plus all non-vertebral fractures;

g original paper reports different numbers for placebo group, correct numbers confirmed by original authors in Avenell 2009;

h hip/wrist/forearm fracture used for non-vertebral fractures;

i unknown how calculated;

j excluded participants receiving intramuscular vitamin D;

k includes participants receiving intramuscular vitamin D;

l osteoporotic fracture;

m fracture numbers calculated indirectly from Table 3 in meta-analysis;

n low trauma fractures;

o low trauma non-vertebral fractures;

p participants with vertebral fracture subtracted from participants with low trauma fractures;

q adjustments to the number of participants with outcomes and denominators were made using an intraclass correlation coefficient of 0.026 to account for cluster randomisation;

r subgroup of participants only;

s hip fractures used for non-vertebral fractures;

t participants with vertebral fractures subtracted from participants with all fractures;

u relative risk only reported for two subgroups,

v total numbers of fractures (not numbers of participants with fracture).

**Table 5:** Reasons for differences in results between meta-analyses, and effects on estimate of efficacy of vitamin D on fracture.

Reason	Effect on estimate of vitamin D efficacy
**Study selection and inclusion**	
• Eligible studies not included	Mixed
• Ineligible studies included	Favourable
**Endpoint definition**	
• Inconsistent approach to endpoint definition	Favourable
(eg data for total fracture, hip fracture, total minus vertebral fracture, or hip/wrist/forearm fracture used inconsistently for non-vertebral fracture)	
• Inconsistent approach to endpoint definition	Favourable
(eg data inconsistently restricted to low trauma fractures)	
**Data extraction**	
• Inconsistent data in original paper not checked with primary authors	Favourable
• Use of data from early timepoint in study	Favourable
(instead of final timepoint)	
• Use of subgroups of participants	Favourable
(instead of data for all randomised participants)	
• Per-protocol analyses	Favourable
(instead of intention-to-treat)	
• Use of adjusted analyses	Favourable
(instead of primary unadjusted intention-to treat analysis)	
• Use of total numbers of fractures not numbers of participants with fractures	Mixed-favourable
**Analytic approaches**	
• Pooling of data for mixed fracture types	Mixed-favourable
(eg hip and total fractures	
• Different approaches to handling data from cluster randomised controlled studies	Neutral

The differences between meta-analyses in relative risks for individual trials were most prominent for the total or non-vertebral fracture endpoint. Non-vertebral fracture has commonly been reported in trials, but is often used interchangeably with total fracture. Some meta-analyses adopted this approach [17], whereas others carried out separate analyses for total fracture and for non-vertebral fracture [18]. In several meta-analyses, there were inconsistent approaches to handling data (Table 3). Participants with hip fracture were added to those with all non-vertebral fracture for 2 trials [25], [31] in one meta-analysis [17], and for one of these two trials [31] in two meta-analyses [16], [19], effectively counting hip fractures twice. In 2 meta-analyses [16], [19], the primary endpoint was non-vertebral fracture but, when this endpoint was not reported, the authors used different estimates of non-vertebral fractures for different individual trials. Thus Table 4 shows that for different trials the authors used total fracture; total fracture minus spine fracture; low trauma fracture minus spine fracture; hip, wrist, or forearm fracture; hip fracture; and counted hip fractures twice. For the 2 trials where only subsets of fractures were used (hip fracture, or hip/wrist/forearm fracture), data on total fractures were available. One meta-analysis utilised one fracture endpoint for each study determined hierarchically in descending order from total fracture, hip fracture, and non-vertebral fracture [21]. For the resulting meta-analysis, total fracture was used for 10 trials, hip fracture for 4 trials, and non-vertebral fracture for 2 trials. Hip fracture is only a small subset of total fracture, and for all 4 trials where hip fracture was used, data on the broader endpoint of non-vertebral fracture were used in other meta-analyses.

Table 6 shows the conclusions from the meta-analysis. Some of the conclusions differ substantially. For example, in 3 meta-analyses Bischoff-Ferrari and colleagues conclude that higher doses but not lower doses of vitamin D prevent fractures [16], [19], [22], whereas 3 other meta-analyses concluded that vitamin D, used without calcium supplements, does not prevent fractures, regardless of the dose [18], [20], [21]. Table 6 shows that our assessment of the strength of the conclusions in favour of vitamin D supplements ranged from mixed to strongly positive, with a median score of ≥4 for 6 of the 7 meta-analyses. The meta-analysis that most closely adhered to the recommended approach to analysis of a randomised controlled trial and fulfilled the most number of items in the AMSTAR tool had the least positive conclusion [18].

**Table 6:** Conclusions of meta-analyses.

Author	Conclusion	Strength of Conclusion (Scale 1–5)^a^	Citations
Bischoff-Ferrari, 2005 [16]	Vitamin D at a dose of 700–800 IU/d but not 400 IU/d reduces risk of hip and non-vertebral fracture	Positive (4.5)	1270
Tang, 2007 [17]	CaD effectively prevents osteoporotic fracture	Positive (4.5)	816
Avenell, 2009 [18]	Vitamin D alone does not prevent fractures. CaD might prevent hip fractures in frail older institutionalised people	Mixed (3)	662
Bischoff-Ferrari, 2009 [19]	Received dose of vitamin D of 482–770 IU/d but not ≤400 IU/d effectively prevents non-vertebral and hip fracture	Positive (4.5)	553
DIPART, 2010 [20]	Vitamin D alone does not prevent fractures. CaD effectively prevents hip and total fractures	Positive (4)	160
Chung, 2011 [21]	CaD but not vitamin D alone can reduce fracture risk. Effects are smaller in community-dwelling than institutionalised individuals	Mixed (4)	146
Bischoff-Ferrari, 2012 [22]	Vitamin D at a dose of ≥800 IU/d prevented hip and non-vertebral fracture.	Positive (5)	205

a Conclusions were rated independently by both authors. There was perfect agreement using the 3-point scale (positive/mixed/negative), and the median value on a 5 point scale is shown. Citations were obtained from Google Scholar in May 2014.

## Discussion

Among overlapping meta-analyses of vitamin D and fracture, there were substantial differences in the trials included, the data used from each trial, the analytical approach adopted, and the conclusions drawn, despite the meta-analyses being of high quality and published in the highest ranking medical journals. Only 4 meta-analyses included all eligible trials, with the number of “missed” trials ranging from 3 to 8 in the other 3 meta-analyses. Two meta-analyses included trials that did not appear to meet eligibility criteria, while excluding other trials of similar design. The relative risks used for individual trials varied between meta-analyses, with differences being more common for total fracture (67% of trials) than for hip fracture (50% of trials). The differences in relative risks led to more favourable estimates of the efficacy of vitamin D compared to analyses using recommended analytic approaches on 11/16 (69%) occasions. The conclusions of the meta-analyses were discordant, ranging from strong statements that vitamin D prevents fractures to equally strong statements that vitamin D used without calcium does not prevent fractures. All meta-analyses were favourable toward prescribing of vitamin D for fracture prevention, although in some meta-analyses the recommendations were restricted to certain subgroups, or to co-administration of vitamin D with calcium supplements.

The reasons for the differences between the meta-analyses for the trials included and the data used (Table 5) were often not readily apparent. An explanation as to why trials were not included was provided in only one meta-analysis. Fracture data for 3 of the trials included in the Cochrane review were unpublished and obtained for that review [18]. It is not clear whether the authors of other meta-analyses sought these unpublished data, or why, once published in the Cochrane review, they were not included in later meta-analyses. On a similar note, the Cochrane review authors clarified ambiguous reporting of treatment group numbers in the primary publication for one study with the lead author of the study [31] (Table 3/4), whereas in the other meta-analyses, incorrect denominators for both treatment groups were used.

The reasons for the differences in relative risks between meta-analyses can only be deduced by detailed, careful examination of the meta-analyses and the primary publications. One trial-level meta-analysis did not report the number of participants with fracture or the number of participants in each treatment group for individual trials [17] and neither patient-level analysis [20], [22] reported these data or relative risks for individual trials. The absence of this information limits verification of the accuracy of the data and analyses undertaken. For patient-level analyses where data is censored at an earlier timepoint [20], this information is very important because the results at the earlier time point may differ from those of the overall trial. For example, in a patient-level meta-analysis of vitamin D and mortality [51], data for the Women's Health Initiative trial [41] was censored at 3 years, restricting the number of deaths to about 25% of those occurring in the trial, and providing a more favourable effect estimate than that for the entire follow-up. Several meta-analyses used data from per-protocol analyses for individual studies. The recommended analysis for a randomised controlled trial is an unadjusted intention-to-treat analysis including all available data from all randomised participants [24]. We think the same principle applies in meta-analyses of randomised controlled trials. When only per-protocol data are reported for trials, the Cochrane handbook recommends performing sensitivity analyses to explore differences between intention-to-treat approaches (that assume participants lost to follow-up did not have an event), with results using per-protocol data [52]. None of the meta-analyses performed such sensitivity analyses. None of the meta-analyses provided sufficient details to permit a reader to understand if data from individual trials could be incorporated in the meta-analysis in different ways (such as using all fractures versus using only low-trauma fractures), and none compared their handling of the data with previous meta-analyses.

The methodological differences between meta-analyses influenced the conclusions drawn from them. Each of the 3 meta-analyses that considered trials of vitamin D with calcium supplements separately to trials of vitamin D [18], [20], [21] concluded that vitamin D alone does not prevent fractures, regardless of dose. However, the 3 meta-analyses by Bischoff-Ferrari and colleagues that assessed vitamin D with or without calcium concluded that higher doses of vitamin D prevent fractures [16], [19], [22]. We have several reservations about the conclusions of these 3 meta-analyses. As highlighted in Tables 3 and 4, these meta-analyses used more favourable effect estimates for vitamin D for individual trials than estimates obtained using unadjusted intention-to-treat analysis of all participants with data from the final study timepoint. Most trials categorised as high dose vitamin D studied co-administered calcium and vitamin D, but the benefits were attributed to vitamin D. Three large trials of high dose vitamin D [40], [45], [50] were excluded or only included in secondary analyses because of their study design, but all had relative risks for fracture with vitamin D greater than 1, essentially excluding clinically significant benefits on fracture prevention for vitamin D (Tables 1,3–4). Finally, 2 of these meta-analyses made questionable assumptions about received vitamin D doses and focused on treatment adherence analyses [19], [22], methodology that has been criticised [53]. In our view, the Cochrane review [18] is the most detailed and comprehensive, receives the highest rating using the AMSTAR tool for quality assessment, includes the broadest range of trials, and utilises the recommended intention-to-treat approach with the most conservative effect estimates. We think the meta-analyses in the Cochrane review are the most reliable with the greatest external validity- i.e. the results are most generalisable to the wider population.

We followed the approach recommended in 1997 for assessment of discordance amongst systematic reviews [9]. The widespread use of checklists for reporting meta-analyses, such as the PRISMA checklist [54], means that later meta-analyses have become more standardised and of higher quality. This is reflected in Table 2 which shows the high level of reporting of AMSTAR items used to assess meta-analysis quality. However, despite the apparent high quality of these meta-analyses, there were important differences in 3 of the domains that give rise to discordant meta-analyses [9]: study selection and inclusion, data extraction, and statistical methods. The differences are not readily apparent unless each meta-analysis is scrutinised in considerable detail. Thus, it is very likely that the casual reader, and even an expert reviewer, will not notice the differences or understand why the results of overlapping meta-analyses differ. Many of the methodological differences are based on subjective decisions made by the authors. Since different researchers make different judgements on these methodological issues, their decisions and the reasoning behind the decisions should be reported. In Table 7, we propose additions to guidelines for the reporting of overlapping meta-analyses to facilitate their interpretation. They may also decrease redundant overlapping meta-analyses [3], by requiring authors to clearly identify previous publications and make apparent what the new meta-analyses adds to existing knowledge. An important limitation of our analysis is that it is limited to a single topic and a sample of meta-analyses published in high-impact journals, but it seems likely that the weaknesses in methodology and reporting we found will be present in other overlapping meta-analyses.

**Table 7:** Suggestions for improved reporting of overlapping meta-analyses.

Abstract	• State article is an overlapping meta-analysis
	• State goal of current meta-analysis
Introduction	• Report number of previous meta-analyses on topic
	• Summarise conclusions of previous meta-analyses
	• State goal of current-meta-analysis
Methods	• Reference all previous meta-analyses on topic
	• List all relevant studies identified in literature search not included in current meta-analysis, and reasons for exclusion
	• State which studies included in previous meta-analyses are excluded from current meta-analysis, and reasons for exclusion
	• State which studies included in current meta-analysis have not been included in previous meta-analyses, and reasons for inclusion
	• State where data for individual studies in current meta-analysis differ from those used in previous meta-analyses, and reasons for differences
	• For patient-level analyses, if data have been censored at an early time point, state whether estimates of effect size differ from estimates at final time point
Results	• Data for numbers of events/participants reported (including for patient-level analyses)
Discussion	• Discuss conclusions of previous meta-analyses
	• Discuss what current meta-analysis adds to existing body of literature
	• If conclusions of current meta-analysis differ from previous meta-analyses, state reasons for differences
Registration	• Mandatory registration of meta-analysis protocol, including statistical analysis plan

There are important clinical consequences arising from discordant conclusions from overlapping meta-analyses. They engender confusion among clinicians and patients, and foster public disenchantment with biomedical research, exemplified by the statement often used in the media that “the experts can't make up their minds”. Another specific possibility is that patients taking vitamin D supplements in the hope of preventing fractures might be falsely reassured that they are improving their skeletal health by the reporting of positive meta-analyses with methodological weaknesses or limited generalisability.

In summary, this detailed review reveals substantial differences between overlapping meta-analyses of vitamin D and fracture published in the highest ranking general medical journals, despite all meta-analyses generally being assessed as high quality using the AMSTAR tool. The reasons for these differences were often not readily apparent, but the differences led to more favourable estimates of the efficacy of vitamin D compared to estimates obtained using recommend analytic approaches. From this specific example, it is possible to propose additional guidelines for reporting meta-analyses, in order to create greater accuracy and transparency, especially amongst overlapping meta-analyses that report discordant results.

## Supporting Information

S1 Appendix List of 24 meta-analyses or systematic reviews of vitamin D and fracture identified in October 2013 search of PubMed using the terms vitamin D; fracture or osteoporosis; systematic review or meta-analysis.
(DOCX)
