# Supplementary material for: A Case Study of Discordant Overlapping Meta-Analyses: Vitamin D Supplements and Fracture
Source: PLoS One. 2014 Dec 31;9(12):e115934. doi: 10.1371/journal.pone.0115934 (PMC4281138; doi:10.1371/journal.pone.0115934)
Supplement: S1 Appendix — List of 24 meta-analyses or systematic reviews of vitamin D and fracture identified in October 2013 search of PubMed using the terms vitamin D; fracture or osteoporosis; systematic review or meta-analysis. (DOCX) [file pone.0115934.s001.docx]

**List of 24 meta-analyses or systematic reviews of vitamin D and fracture identified in October 2013 search of PubMed using the terms vitamin D; fracture or osteoporosis; systematic review or meta-analysis.**

1: Bischoff-Ferrari HA, Willett WC, Orav EJ, Lips P, Meunier PJ, Lyons RA, Flicker L, Wark J, Jackson RD, Cauley JA, Meyer HE, Pfeifer M, Sanders KM, Stähelin HB, Theiler R, Dawson-Hughes B. A pooled analysis of vitamin D dose requirements for fracture prevention. N Engl J Med. 2012;367:40-9.

2: Murad MH, Drake MT, Mullan RJ, Mauck KF, Stuart LM, Lane MA, Abu Elnour NO, Erwin PJ, Hazem A, Puhan MA, Li T, Montori VM. Clinical review. Comparative effectiveness of drug treatments to prevent fragility fractures: a systematic review and network meta-analysis. J Clin Endocrinol Metab. 2012;97:1871-80.

3: Chung M, Lee J, Terasawa T, Lau J, Trikalinos TA. Vitamin D with or without calcium supplementation for prevention of cancer and fractures: an updated meta-analysis for the U.S. Preventive Services Task Force. Ann Intern Med. 2011;155:827-38.

4: Bergman GJ, Fan T, McFetridge JT, Sen SS. Efficacy of vitamin D3 supplementation in preventing fractures in elderly women: a meta-analysis. Curr Med Res Opin. 2010;26:1193-201.

5: DIPART (Vitamin D Individual Patient Analysis of Randomized Trials) Group. Patient level pooled analysis of 68 500 patients from seven major vitamin D fracture trials in US and Europe. BMJ. 2010;340:b5463.

6: Lai JK, Lucas RM, Clements MS, Roddam AW, Banks E. Hip fracture risk in relation to vitamin D supplementation and serum 25-hydroxyvitamin D levels: a systematic review and meta-analysis of randomised controlled trials and observational studies. BMC Public Health. 2010;10:331.

7: Avenell A, Gillespie WJ, Gillespie LD, O'Connell D. Vitamin D and vitamin D analogues for preventing fractures associated with involutional and post-menopausal osteoporosis. Cochrane Database Syst Rev. 2009;CD000227.

8: Bischoff-Ferrari HA, Willett WC, Wong JB, Stuck AE, Staehelin HB, Orav EJ, Thoma A, Kiel DP, Henschkowski J. Prevention of nonvertebral fractures with oral vitamin D and dose dependency: a meta-analysis of randomized controlled trials. Arch Intern Med. 2009;169:551-61.

9: Parikh S, Avorn J, Solomon DH. Pharmacological management of osteoporosis in nursing home populations: a systematic review. J Am Geriatr Soc. 2009;57:327-34.

10: MacLean C, Newberry S, Maglione M, McMahon M, Ranganath V, Suttorp M, Mojica W, Timmer M, Alexander A, McNamara M, Desai SB, Zhou A, Chen S, Carter J, Tringale C, Valentine D, Johnsen B, Grossman J. Systematic review: comparative effectiveness of treatments to prevent fractures in men and women with low bone density or osteoporosis. Ann Intern Med. 2008;148:197-213.

11: Reid IR, Bolland MJ, Grey A. Effect of calcium supplementation on hip fractures. Osteoporos Int. 2008;19:1119-23.

12: Boonen S, Lips P, Bouillon R, Bischoff-Ferrari HA, Vanderschueren D,Haentjens P. Need for additional calcium to reduce the risk of hip fracture with vitamin d supplementation: evidence from a comparative metaanalysis of randomized controlled trials. J Clin Endocrinol Metab. 2007;92:1415-23.

13: Cranney A, Horsley T, O'Donnell S, Weiler H, Puil L, Ooi D, Atkinson S, Ward L, Moher D, Hanley D, Fang M, Yazdi F, Garritty C, Sampson M, Barrowman N, Tsertsvadze A, Mamaladze V. Effectiveness and safety of vitamin D in relation to bone health. Evid Rep Technol Assess (Full Rep). 2007;1-235.

14: Izaks GJ. Fracture prevention with vitamin D supplementation: considering the inconsistent results. BMC Musculoskelet Disord. 2007;8:26.

15: Jackson C, Gaugris S, Sen SS, Hosking D. The effect of cholecalciferol (vitamin D3) on the risk of fall and fracture: a meta-analysis. QJM. 2007;100:185-92.

16: Oliver D, Connelly JB, Victor CR, Shaw FE, Whitehead A, Genc Y, Vanoli A, Martin FC, Gosney MA. Strategies to prevent falls and fractures in hospitals and care homes and effect of cognitive impairment: systematic review and meta-analyses. BMJ. 2007;334:82.

17: Tang BM, Eslick GD, Nowson C, Smith C, Bensoussan A. Use of calcium or calcium in combination with vitamin D supplementation to prevent fractures and bone loss in people aged 50 years and older: a meta-analysis. Lancet. 2007;370:657-66.

18: Nakamura K, Iki M. Efficacy of optimization of vitamin D in preventing osteoporosis and osteoporotic fractures: A systematic review. Environ Health Prev Med. 2006;11:155-70.

19: Whelan AM, Jurgens TM, Bowles SK. Natural health products in the prevention and treatment of osteoporosis: systematic review of randomized controlled trials. Ann Pharmacother. 2006;40:836-49.

20: Bischoff-Ferrari HA, Willett WC, Wong JB, Giovannucci E, Dietrich T, Dawson-Hughes B. Fracture prevention with vitamin D supplementation: a meta-analysis of randomized controlled trials. JAMA. 2005;293:2257-64.

21: Richy F, Schacht E, Bruyere O, Ethgen O, Gourlay M, Reginster JY. Vitamin D analogs versus native vitamin D in preventing bone loss and osteoporosis-related fractures: a comparative meta-analysis. Calcif Tissue Int. 2005;76:176-86.

22: Häuselmann HJ, Rizzoli R. A comprehensive review of treatments for postmenopausal osteoporosis. Osteoporos Int. 2003;14:2-12.

23: Papadimitropoulos E, Wells G, Shea B, Gillespie W, Weaver B, Zytaruk N, Cranney A, Adachi J, Tugwell P, Josse R, Greenwood C, Guyatt G; Osteoporosis Methodology Group and The Osteoporosis Research Advisory Group. Meta-analyses of therapies for postmenopausal osteoporosis. VIII: Meta-analysis of the efficacy of vitamin D treatment in preventing osteoporosis in postmenopausal women. Endocr Rev. 2002;23:560-9.

24: Vallecillo G, Díez A, Carbonell J, González Macías J. [Treatment of osteoporosis with calcium and vitamin D. Systematic review]. Med Clin (Barc). 2000;115:46-51.

**Superseded reviews**

25. Chung M, Balk EM, Brendel M, Ip S, Lau J, Lee J, Lichtenstein A, Patel K, Raman G, Tatsioni A, Terasawa T, Trikalinos TA. Vitamin D and calcium: a systematic review of health outcomes. Evid Rep Technol Assess (Full Rep). 2009;1-420.

26: Avenell A, Gillespie WJ, Gillespie LD, O'Connell DL. Vitamin D and vitamin D analogues for preventing fractures associated with involutional and post-menopausal osteoporosis. Cochrane Database Syst Rev. 2005;CD000227.

27. Gillespie WJ, Avenell A, Henry DA, O'Connell DL, Robertson J. Vitamin D and vitamin D analogues for preventing fractures associated with involutional and post-menopausal osteoporosis. Cochrane Database Syst Rev. 2001;CD000227.

28. Gillespie WJ, Henry DA, O'Connell DL, Robertson J. Vitamin D and vitamin D analogues for preventing fractures associated with involutional and post-menopausal osteoporosis.

Cochrane Database Syst Rev. 2000;CD000227.
